# Developmental and maintenance defects in Rett syndrome neurons identified by a new mouse staging system *in vitro*

**DOI:** 10.3389/fncel.2014.00018

**Published:** 2014-02-05

**Authors:** Gabriele Baj, Angela Patrizio, Alberto Montalbano, Marina Sciancalepore, Enrico Tongiorgi

**Affiliations:** Department of Life Sciences, BRAIN Center for Neuroscience, University of TriesteTrieste, Italy

**Keywords:** Rett Syndrome, neurodevelopment, hippocampal neurons, Dendritic Spines, Dendrites, staging system, Neuronal morphology

## Abstract

Rett Syndrome (RTT) is a neurodevelopmental disorder associated with intellectual disability, mainly caused by loss-of-function mutations in the *MECP2* gene. RTT brains display decreased neuronal size and dendritic arborization possibly caused by either a developmental failure or a deficit in the maintenance of dendritic arbor structure. To distinguish between these two hypotheses, the development of *Mecp2*-knockout mouse hippocampal neurons was analyzed *in vitro*. Since a staging system for the *in vitro* development of mouse neurons was lacking, mouse and rat hippocampal neurons development was compared between 1–15 days *in vitro* (DIV) leading to a 6-stage model for both species. *Mecp2*-knockout hippocampal neurons displayed reduced growth of dendritic branches from stage 4 (DIV4) onwards. At stages 5–6 (DIV9-15), synapse number was lowered in *Mecp2*-knockout neurons, suggesting increased synapse elimination. These results point to both a developmental and a maintenance setback affecting the final shape and function of neurons in RTT.

## Introduction

Rett Syndrome (RTT) is an X-linked neurodevelopmental disease associated with intellectual disability. RTT's main cause are mutations in the *MECP2* gene that encodes a transcriptional regulator (Amir et al., 1999). Postmortem studies revealed a decrease in neuronal size and dendritic arborization in the brain of RTT individuals with increased cell density and global reduction in brain volume (Belichenko et al., 1994, 2009; Subramaniam et al., 1997; Kaufmann and Moser, 2000; Armstrong, 2001; Akbarian, 2002; Saywell et al., 2006). The pathological process leading to these features is still unclear and recent studies in animal models of RTT using conditional *Mecp2* knock-out in adult mice have challenged the idea of a developmental failure by suggesting the hypothesis of a deficit in the maintenance of the dendritic arbor structure (Schule et al., 2008; Matijevic et al., 2009).

Neuronal development is a complex multistep process that can be divided in several, partially overlapping stages (Dotti et al., 1988; Kossel et al., 1997; Wu et al., 1999; Scott and Luo, 2001; Urbanska et al., 2008; Ehlers and Polleux, 2010). One efficient approach to study neurodevelopmental deficits in disorders such as Down, RTT and Fragile-X syndromes, has been the analysis of neuronal maturation *in vitro* (Saud et al., 2006; Barnes and Polleux, 2009; Jacobs et al., 2010; Kim et al., 2011; Gleeson and Polleux, 2012). The most accurate description of neuronal development *in vitro* available to date, refers to rat hippocampal neurons and was published in 1988 by Dotti et al. (1988). They identified five main stages and annotated the time, up to 7 days *in vitro* (DIV), at which hippocampal neurons enter each stage. The Dotti model represents the most used staging system for rat hippocampal neurons, but recent studies highlighted additional features that were not described in the original study (Horton et al., 2006; Kaech and Banker, 2006; Urbanska et al., 2008). For instance, Horton et al. (2006) showed that the first 5 stages during which axons and dendrites grow and elongate, are followed by a dynamic phase, at DIV8-10, during which one dendrite presents a high level of retractions and protrusions to become the apical dendrite, preceding a final stabilization phase that occurs over a longer time period (Horton et al., 2006; Barnes and Polleux, 2009). These studies suggested the need to extend beyond DIV7 the current *in vitro* staging model for the rat hippocampal neurons development. Other systems, like organotypic cultures or brain slices, can represent an excellent compromise between single cell cultures and whole animal studies, replacing and reducing the number of animal experiments (Mewes et al., 2012). However their usage on large scale remains limited due to their cost, miniaturization limits, and complexity (Sharma et al., 2012). Considering that many mouse models of neurological diseases have been created, the availability of a staging model for primary mouse neurons would represent a major tool to investigate the pathological processes in these diseases. Accordingly, this study aims at revising the current staging system for rat neurons and developing the staging system for the development of mouse hippocampal neurons *in vitro* to identify which steps in the maturation process are impaired in RTT.

## Materials and methods

### Primary cultures of rat hippocampal neurons

Animal use was approved by the Italian Ministry of Health under authorization n°185/2010-B. Primary hippocampal neurons were prepared from postnatal day 1 rats as described by Aibel et al. (1998), with slight modifications. Cells were plated on 2% Matrigel (BD Biosciences)-coated coverslips in 24-well plates at a density of 4 × 10^5^ cells/mL per well and cultured in a 5% CO_2_ humidified incubator in Neurobasal media (Invitrogen) supplemented with B27 (Invitrogen), 1 mM L-glutamine (Euroclone), and antibiotics (Euroclone). Cell number was assessed by a dye exclusion method using Trypan Blue (Fluka) and cells were plated at density of 600–800 cells/mm^2^. The medium was changed every 2 days from the second day in culture onward. Cell density was recorded at specific time points DIV3, 6, 9, 12, and 15 in order to verify the culture reproducibility.

### Primary cultures of mouse hippocampal neurons

Wild-type C57/BL6 mice were purchased from Charles River Laboratories (Calco, LC, Italy). Female *Mecp2* heterozygous mice (Guy et al., 2001) were purchased from Jackson Laboratories (Bar Harbor, Maine; strain name: B6.129P2(C)-MeCP2tm1.1Bird/J, stock number: 003890). *Mecp2* knockout mice were obtained by crossing *Mecp2* heterozygous females with wild type C57/BL6 males. Hippocampal neurons were prepared from postnatal day 2 (P2) knockout mice (males) and WT pups using the procedure previously described. In this case, hippocampi from each mouse were dissected and plated separately from those of the other littermates in order to isolate knockout neurons from WT neurons. In all cell cultures, growth of non-neuronal cells was prevented by adding 5.0 uM cytosine b-D-arabinofuranoside (ARA-C) on the second day in culture. Cells were maintained *in vitro* from 2 to 15 days (2–15 DIV) at 37°C in a 5% CO_2_-humified incubator.

### Mouse genotyping (B6.129P2(C)-MeCP2tm1.1BIRD/J)

Mouse genotype was identified by PCR following the extraction of genomic DNA from tails. Two different mixes with a specific reverse primer to either amplify the mutant or the wild type form of *Mecp2* gene were prepared (reverse mutant primer oIMR1437 5′- TCC ACC TAG CCT GCC TGT AC -3′, reverse wild type primer oIMR1438 5′- GGC TTG CCA CAT GAC AA -3′), and a forward common primer (oIMR1436 5′- GGT AAA GAC CCA TGT GAC CC -3′).

### Cell transfection

Neuron transfection was performed using Lipofectamine 2000 (Life Technologies) following manufacturer instructions. In brief, 2 μg of pEGFP-N1 vector (Clontech) was used for each well and this transfection mix was removed after 1 h. Cells were transfected at 1, 3, 5, 7, 9, 11, 13 DIV, 24–48 h prior to fixation and immunofluorescence for morphology measurements.

### Immunofluorescence

Immunostaining of cultured hippocampal neurons was performed with the following procedure. Cells were fixed in 4% paraformaldehyde (PFA) in phosphate buffer saline (PBS) for 20 min and washed in PBS. After a preliminary permeabilization with PBS with 0.1% Triton (PBST) for 30′, cells were incubated for 2 h at room temperature with the primary antibody in 5% normal goat serum in PBST. After washes in PBST, cells were incubated 1 h at room temperature with the respective secondary anti-mouse or anti rabbit IgG antibodies Alexa 488, Alexa 568 or Alexa 640 (1:250; Invitrogen) in 2% normal goat serum in PBST. Finally, cells were washed in PBST and stained with the nuclear stain Höechst and the coverslips were mounted in Mowiol antifade compound (Sigma). The following antibodies were used: Rabbit polyclonal anti-MAP2 (Santa Cruz Biotechnology); Mouse monoclonal anti-MAP2 (Sigma); Mouse monoclonal anti-Tau-1 (Santa Cruz Biotechnology); Mouse monoclonal anti-PDS95 (Millipore); Mouse monoclonal anti-Gephyrin (SySy, kindly provided by Prof. Triller); Rabbit polyclonal anti Synapsin-1 (Millipore); anti-mouse Alexa Fluo 647 (Invitrogen); anti-rabbit Alexa Fluo 568 (Invitrogen); anti-mouse Alexa Fluo 488 (Invitrogen).

### Microscopy

**Figure 1:**
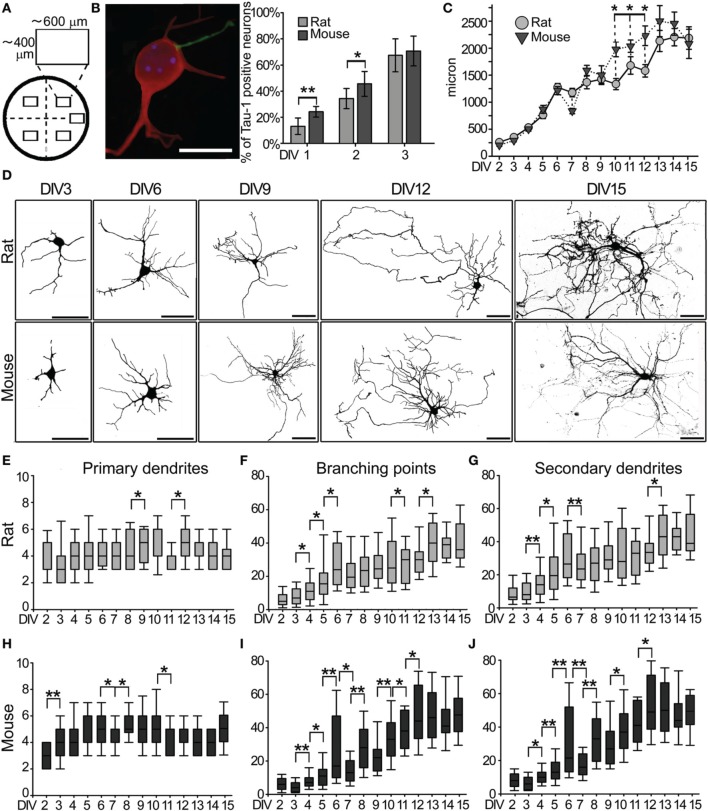
**Morphological characterization of rat and mouse hippocampal neurons**. **(A)** Neurons were counted from five sampling areas (rectangles) per coverslip. **(B)** DIV2 mouse hippocampal neuron immunostained for MAP2 (red) and Tau-1 (green) and quantification (mean + s.e.m.) of the percentage of neurons with one Tau-1 positive/MAP2-negative neurite in mouse and rat neuronal cultures. (Scale Bar = 10 μm) **(C)** Quantitative data of the average total dendritic length of mouse and rat hippocampal neurons *in vitro*. Data are expressed as mean ± s.e.m., *n* = 50. **(D)** Images of GFP-transfected rat and mouse hippocampal neurons at the indicated DIV (Scale Bar = 50 μm). **(E)** Number of primary dendrites, **(F**) branch points and **(G**) secondary dendrites in rat neurons at each DIV. **(H)** Primary dendrites, **(I)** branch points and **(J)** secondary dendrites in mouse neurons. The median line of the boxes refers to the 50 percentile of the entire population (*N* = 50 per DIV). Mann-Whitney Rank Sum Test, ^**^P ≤ 0.01, ^*^P ≤ 0.05.

Digital images of GFP-positive neurons, with MAP2 staining and nuclear Höechst staining, as well as those of GFP-negative neurons (i.e., not transfected with pEGFP-N1) stained against MAP2, Tau-1 and with Höechst staining, were acquired using a Nikon Eclipse E800 epifluorescence microscope with a 20X objective and a Nikon DXM1200 camera, paired with ACT-1 software. All the 12 mm^2^ glass coverslips (Sacco) used to culture cells have been divided in four squared fields that have been considered in order to take pictures in the most reproducible manner possible (see Figure 1A and Results). Dendritic spines were imaged in a Nikon C1si confocal microscope equipped with an argon laser (457, 477, 488, and 514 nm lines), a 561 nm diode laser and a 640 nm diode laser. Excitation light was delivered to the sample with an 80/20 reflector. To avoid any possible cross-talk phenomena among fluorophores (“bleed trough”), all images were collected using the method of sequential line scanning. The system was operated with a pinhole size of Airy disk (30 nm) (for spines images the pinhole was set 60 nm). A 60X Oil Apo objective (NA 1.4) was used, collecting series of optical images at 0.15 μm z resolution (step size), which gave a voxel size of 75 × 75 × 150 nm (X × Y × Z). Images were then processed for z-projection using ImageJ 1.45 (NIH, Bethesda, USA). We collected images of both proximal and distal fields of the apical dendrite of neurons. Quantitative data were collected considering the average intensity of the signal coming from all the stacks of a single image.

### Tau-1 analysis

The presence of the axon was determined in GFP-negative cells (i.e., not transfected with pEGFP-N1) fixed with PFA 4% at DIV1, 2, and 3 respectively. To define the percentage of cells that present the axon at each time point, 5 different fields (see Results, Figures 1A,B) from two different coverslips have been analyzed as follows: first, MAP2-positive cells were counted in order to discriminate between neurons and glial cells, and among these, the number of axons (Tau-1 positive and MAP2- negative neurites) was established.

### Polarization analysis

**Figure 2:**
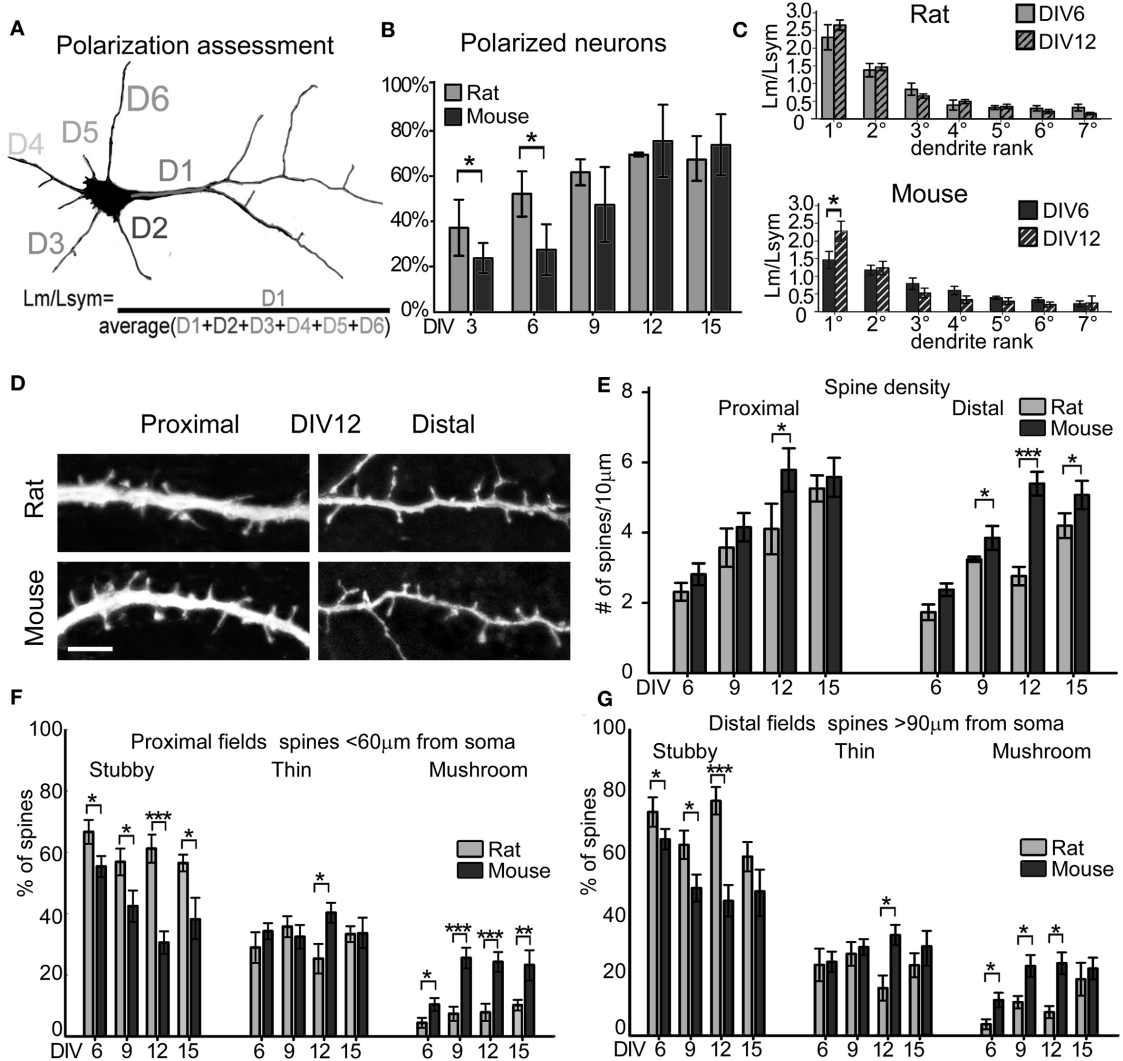
**Dendrites and spines maturation. (A)** The length of the longest dendrite D1 (Lm) is divided by the average length of all dendrites D1, D2, D3, D(n) (Lsym). The dendritic arbor is polarized when the apical dendrite is specified, i.e., when Lm/Lsym = 2. **(B)** Percentage of neurons with polarized dendritic arbor in rat and mouse cultures at different DIV. Mann-Whitney Rank Sum Test, ^*^P = 0.05. **(C)** Contribution of each dendrite to total dendritic length at DIV6 and DIV12, expressed as the ratio of measured dendrite length (Lm) to the Lsym. Dendrites are ranked longest to shortest (*N* = 50 neurons; 3–4 cultures). **(D)** Proximal and distal 40 μm-long fields of mouse and rat apical dendrites. (Scale Bar = 5 μm). **(E)** Spine density in apical dendrite proximal and distal region of mouse (dark gray) and rat (light gray) at DIV6, 9, 12, 15. **(F,G)** Spine type percentage (mean, standard errors). ^***^p ≤ 0.001, ^**^p ≤ 0.01, ^*^p ≤ 0.05 (One-Way ANOVA with Holm-Sidak's pairwise multiple comparison).

To define the degree of polarization of neuronal dendrites, we followed the method described by Horton et al. (2006). Briefly, the measured lengths of dendrites (Lm) in each neuron (including the primary dendrite with all its branches) were ranked highest to lowest. The sum of these lengths is the total dendritic length for that neuron, and the proportion contributed by each dendrite to that total is: Lm/Lsym = Length of dendrite/Total dendritic length. If neurons were symmetric (i.e., with dendrites of similar lengths), that value would be *Lm* = Lsym = 1 / number of dendrites (Lm/Lsym = 1). If neurons were asymmetric (i.e., with dendrites of different lengths), the ratio Lm/Lsym would reflect the degree to which dendrites diverge from perfect symmetry. All neurons that showed a Lm/Lsym value above a threshold of 2 were considered as polarized neurons. To quantify the contribution of each dendrite to the total dendritic length in a cell-by-cell basis, dendrites were ranked on the basis of the ratio between their lengths (Figure 2C; Lm) divided by the mean length of all dendrites from the same neuron (Figure 2C; Lsym).

### Morphological analysis

The morphological analysis of hippocampal neurons was focused on 4 different parameters: total dendritic length, number of primary and higher order dendrites, and number of branch points. To achieve this aim, dendritic length and branch points of all GFP-positive neurons were measured and quantified by tracing along each neuronal projection using ImageJ software (NIH, Bethesda, USA). The starting point of a dendrite was defined as the point at the midline of the dendrite that intersected the curvature of the soma. For our measures, protrusions emerging from the cell soma with all its branches were counted as a single dendrite, tracing the entire dendritic arbor before moving to measure other primary dendrites (Figure 2A). The branch points were counted using the Multi-point tracker plugin. Filopodia protrusions (considered as >5 microns-long), were excluded from the branch point analysis.

### Morphological analysis of dendritic spines

Spines are usually classified as stubby, thin, and mushroom based on the morphological criteria proposed by Harris et al. (Harris et al., 1992). In particular, they are defined as stubby if their neck diameter and their total length are comparable, as thin if their length was much greater than both the neck and head diameters (which are similar), and as mushroom if the head diameter was much larger than the neck diameter (cup-shaped spines were considered as mushroom). Following a visual classification based on known morphological criteria, the Straight-line tool of ImageJ software was used to measure spine length (traced from the edge of the dendrite to the tip of the spine head) and spine head diameter, and as well as dendrite length used to determine spine density. Dendritic length was also evaluated in order to determine spine density in both proximal and distal fields of the apical dendrite (Smith et al., 2009). Our measurements come from 800 μm of total length per region, collected from at 12–15 neurons.

### Co-localization of Synapsin-1 and PSD-95

The degree of SYN1 and PSD95 or Gephyrin co-localization with SYN1 was evaluated from confocal images. Quantification of co-localized pixels was performed using the Colocalization module in Imaris software (Bitplane). Imaris was used in a default setting with the addition of an automatic threshold for pixel intensity calculated using the point spread function measured in our microscope. Colocalization was quantified as amount of fluorescence collected from each pixel stained (over the threshold) for both protein targets. In brief, the volumetric percentage of signal (IMARIS) over the threshold for these synaptic proteins is the number used as co-localization amount.

### Electrophysiology

Whole-cell current-clamp recordings were performed in single neurons at room temperature from both groups of animals (mice and rats). Cells were continuously perfused (2 mL/min) with normal external solution (NES) containing (in mM): NaCl 145, KCl 4, CaCl_2_ 2, N-2-hydroxyethylpiperazine-N-2-ethanesulfonic acid (HEPES) 10, Glucose 10. Patch pipettes had a resistance of 4–6 MΩ when filled with an intracellular solution containing the following (in mM): potassium-gluconate 135, HEPES 10, NaCl 10, Mg_2_-ATP 2, Na^+^-GTP 0.3; adjusted to pH 7.3 with KOH. Neurons were visualized using a Zeiss microscope (Model Axiovert100). Data were acquired with an Axopatch 200-B amplifier (Molecular Devices) and digitized with a Digidata-1231 (Molecular Devices) computer interface and PClamp 8.0 (Molecular Devices) acquisition software. Signals were sampled at 33 kHz and low-pass filtered at 2 kHz. The stability of the whole-cell configuration was checked by continuously monitoring the input and series resistances during the experiments. Only stable, long-lasting recordings were used for analysis. Cells with a change in leak currents of more than 10% were rejected from the analysis.

### Methodological considerations

Despite the high variability among culturing methods described in the literature, several parameters of the culture conditions and staging system proposed here are found to be conserved between different published protocols: (i) similar percentage of GABAergic neurons, i.e., 20% GAD65/67 positive neurons in both our rats (P0-1 pups) and Horton et al. (E18) cultures (Horton et al., 2006); (ii) conserved gross morphology and conserved occurrence of all the stages [with respect to Dotti's stages 1–5 (Dotti et al., 1988)] with all methods used, independent of age (P0-1 or E18) and irrespective of the type of surface coating and culture medium (Dotti et al., 1988; Bacci et al., 1999; Horton et al., 2006); (iii) similar onset of spontaneous network activity (Bacci et al., 1999); (iv) similar density and type of spines; (v) similar onset of dynamic phase with neurons from P0-1 and E18 (this study, Horton et al., 2006). The morphological analysis was performed on pictures collected from randomly transfected neurons to achieve an un-biased measurement of neurodevelopment *in vitro*. The electrophysiological study was based specifically on pyramidal neurons where their triangular shape and apical dendrite were clearly visible, therefore easily distinguishable from bipolar or multipolar non-pyramidal cells.

### Data representation and statistical analysis

In the bar graphs, columns represent the mean of all measurements (see Results) with corresponding standard errors of the mean (s.e.m.). All statistical data analysis and data plotting were performed using the Prism 5 software (Graphpad). Statistical significance for comparisons between different groups was established using either a Student *t*-test, or Two-way and One-Way ANOVA followed by an all pairwise multiple comparison procedure (Holm-Sidak's method) where ^***^p ≤ 0.001, ^**^p ≤ 0.01, and ^*^p ≤ 0.05. In presence of data not passing the normality test (Shapiro-Wilk) the statistical significant difference was calculated using Mann-Whitney Rank Sum Test where ^***^p ≤ 0.001, ^**^p ≤ 0.01, and ^*^p ≤ 0.05. The different statistical tests are reported in figure legends.

## Results

Since a staging system for hippocampal mouse neuron development *in vitro* was not available, we started by creating a new one and we compared it with the development of rat hippocampal neurons *in vitro*. This strategy was chosen in order to relate this novel mouse staging system with the body of the available information, which was related to the rat, only. A complete analysis of the development of rat and mouse hippocampal neurons DIV1 to DIV15 was performed by imaging neuronal morphology following transfection with a plasmid to express the green-fluorescent protein (GFP). Cultures from both species showed reproducible morphological parameters when plated in the range of 600–800 cells/mm^2^. The percentage of GAD65/67-positive neurons at DIV6 was 18.4 ± 10.6% in rat and 21.6 ± 10.3% in mouse cultures. Hence, the large majority of neurons in our cultures is represented by pyramidal neurons. Accordingly, this study was focused on pyramidal neurons which show very distinctive morphology by DIV4 onward and therefore can be selected on the basis of morphological criteria. Due to the low number of GABAergic interneurons, neurons selected for the quantitative analysis are likely to be true pyramidal neurons even at early stages when these morphological criteria are less stringent.

### First days *in vitro*: axonal specification

Dotti et al. (1988) showed that during the first 2 DIV one neurite is committed to become an axon. We quantified the proportion of rat and mouse neurons with a differentiated axon, identified as a neurite positive for the axonal marker Tau-1, and negative for the dendritic marker MAP2 (Gordon-Weeks, 1991; Hirokawa et al., 1991; Matus, 1991; Hirokawa, 1994; Mandell and Banker, 1996) (Figures 1A,B). Rat neurons with a Tau-1 positive/MAP2 negative neurite were 13 ± 6% at DIV1 and 34 ± 8% at DIV2, while they were 21 ± 4% at DIV1 and 43 ± 9% at DIV2 in mouse cultures (Figure 1B). Even if more mouse neurons showed a faster axonal specification at DIV1 and 2 than rat neurons, a comparable percentage of neurons with specified axons was found in rat (67 ± 12%) and mouse (71 ± 11%) cultures by DIV3 (Figure 1B). These results extend the timing for axonal specification to DIV3.

### Morphological development of dendrites

The total dendritic length was compared between rat and mouse GFP-transfected neurons from DIV2 to DIV15 (Figures 1C,D). Dendrites of neurons from both species showed a comparable progressive increase in their total length up to DIV6, followed by a plateau phase around DIV7-9, and a further progressive increase from DIV10 to DIV15 (Figure 1C). However, between DIV10-12, mouse neurons showed a significantly greater increase in total dendritic length compared to rat neurons, reaching a comparable total dendritic length by DIV15 (mouse 2,066 ± 168 μm; rat 2,173 ± 107; *P* = 0.752). The same neurons were further considered to study the development of primary and secondary dendrites, and their branch points at each DIV (Figures 1E–J). After an initial phase spanning DIV1-3 characterized by the production of primary neurites, the number of primary dendrites stabilized at 4–5 per neurons in both mouse and rat cultures for all subsequent DIVs (Figures 1E,H). Quantification of branch points and secondary dendrites revealed an initial slow rise in the number of branches from DIV2 to DIV6 in both species, followed by an unstable phase with a large variability at DIV6 and a decrease at DIV7 which was particularly strong in mouse cultures (Figures 1F,G for rat, Figures 1I,J for mouse neurons). In rat neurons, the number of dendritic branch points and secondary dendrites remained substantially unchanged between DIV7 and DIV11 (with one variation at DIV10-11 for the number of branch points), while mouse neurons showed an extended unstable phase of alternating expansions and regressions between DIV7 and DIV9. This process culminated in both species with a robust increase in the number of branch points and secondary dendrites between DIV10-12 for mouse neurons and DIV12-DIV13 for rat neurons. The final phase from DIV13 to DIV15 was characterized by a stabilization of the dendritic arbor. In general, morphometric parameters of mouse neurons showed higher s.e.m. values than rat neurons, indicating a broader phenotypical distribution (Figures 1E–J).

### Specification of the apical dendrite

To determine the time point at which rat and mouse neurons specify their apical dendrite, we evaluated GFP-expressing neurons in cultures at DIV6, 9, 12, and 15. We followed the quantitative methodology described by Horton et al. (2006), which identifies the apical dendrite as the one having a total length longer than 2-fold the average dendritic length (Figure 2A). Apical dendrite maturation occurs when D1 length (Lm) doubles the average dendritic length (Lsym), in contrast, when the ratio Lm/Lsym approaches 1, the dendritic arbor is symmetric, like in immature excitatory neurons or mature GABAergic inhibitory neurons. Neuronal cultures are defined as “polarized” when the percentage of neurons with an apical dendrite reaches 60% of the total number of neurons. In our hands, this threshold was reached at DIV9 in rat cultures (62.1% ± 5.77) and DIV12 in mouse cultures (76% ± 16.07). Rat cultures were more polarized compared to mouse cultures at DIV3 and 6 even though they had comparable total dendritic lengths (Figure 1C), and reached similar polarization values at later stages (Figure 2B). In mouse cultures, the ratio Lm/Lsym (see Methods) for the 1st ranked dendrite (i.e., the apical dendrite) passed the threshold of 2.0 at DIV12 (Lm/Lsym = 2.34 ± 0.12), while it was already 2.2 ± 0.28 at DIV6 in rat cultures, reaching 2.6 ± 0.09 by DIV12 (Figure 2C). For 2nd to 7th rank dendrites, the ratio Lm/Lsym remained stable between DIV6 and 12 in both species (Figure 2C).

### Density and morphology of dendritic spines

**Figure 3:**
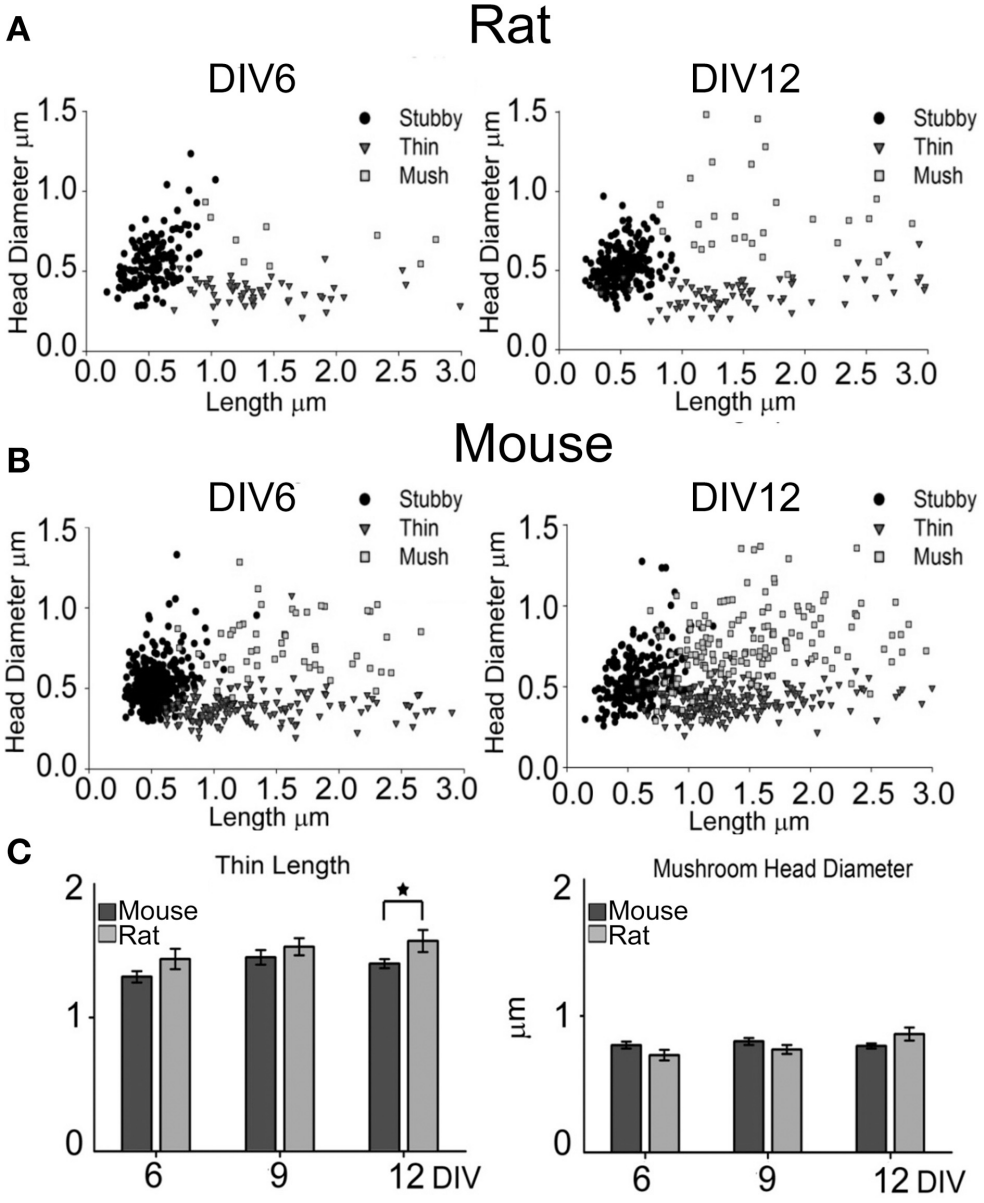
**Spine measurements and categorization at DIV6 and DIV12**. Scatter plots representation for rat **(A)** and mouse **(B)** neurons. X-axis represents the length of each spine, while the y-axis shows the head diameter. Sorting of spine type was performed by operator during measurements; circle: stubby, triangle: thin and square: mushroom spines. (*N* = 15 fields from different neurons from 3–4 different cultures). **(C)** Average length (±SE) of thin spines and average (±SE) head diameter of mushroom spines at DIV6, 9 and 12 for mouse (dark gray bars) and rat (light gray bars) neurons. Student *t*-test ^*^P ≤ 0.05.

To analyze the formation of dendritic spines, we estimated dendritic spine density by counting the number of spines per 10 μm of dendritic length in GFP-expressing neurons at DIV6, 9, 12, and 15. This analysis was carried out on apical dendrites, collecting separately the number of spines in the primary dendrite (proximal) and in secondary dendrites (distal; Figures 2D,E). Spine density in rat cultures increased progressively from DIV6 to DIV15 in both primary and secondary dendrites, while mouse neurons reached their highest spine density already by DIV12. A comparison between the species revealed a significantly higher spine density in secondary dendrites of mouse neurons at DIV9 (mouse 3.84 ± 0.33 spines/10 μm vs. rat 3.23 ± 0.1 spines/10 μm; *t*-test *P* = 0.043), DIV12 (mouse 5.3 ± 0.34 spines/10 μm vs. rat 2.8 ± 0.26 spines/10 μm; *t*-test *P* ≤ 0.001) and DIV15 (mouse 5.0 ± 0.3 spines/10 μm vs. rat 4.1 ± 0.37 spines/10 μm; *t*-test *P* = 0.047). Spines were classified into the three main morphological types: stubby, thin and mushroom (Harris et al., 1992), and the percentage of each spine type was calculated in primary and secondary dendrites (Figures 2F,G). Primary and secondary dendrites of mouse neurons showed a progressive decrease in the percentage of stubby spines from DIV6 to DIV15, while mushroom spines increased from DIV6 to 9, remaining at the same level between DIV12 and 15 (DIV6 vs. 12 ANOVA *P* = 0.005; DIV12 vs.15 ANOVA *P* = 0.4). In contrast, rat neurons showed no changes in the proportions of all spine types in primary dendrites from DIV6 to 15, while there was a decrease of stubby spines between DIV12 and 15 accompanied by an increase in mushroom spines in secondary dendrites (ANOVA, *P* = 0.027). The proportion of thin spines remained constant in both species throughout these *in vitro* time points. To verify if the assignment to the three spine categories was correct, the length and head diameter of each spine were measured (Figure 3). Scatter plots showed a remarkable distinction of the three spine types in three subpopulations and a shift toward a higher number of large mushroom spines is evident between DIV6 to DIV12 (Figures 3A,B). Analysis of the average length of thin spines and of the average head diameter of mushroom spines showed no differences between DIV6 and DIV12 in both species (Figure 3C). Interestingly, thin spines in mouse neurons were shorter than those in rat neurons (*P* = 0.026), while other dimensions were comparable.

### Synaptogenesis

**Figure 4:**
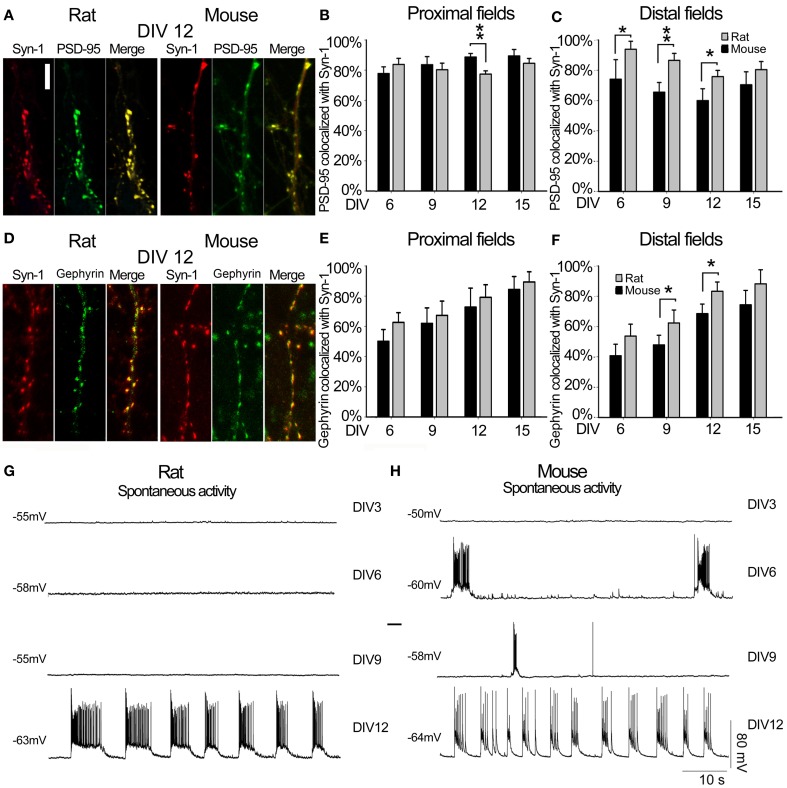
**Synapse characterization**. **(A)** Representative image of SYN1 (red panel) and PSD95 (green panel) signals, and co-localization (merge panel) at DIV12 in rat and mouse hippocampal neurons (Scale bar = 5 μm). **(B)** Quantitative data representing the degree of co-localization between the two synaptic proteins Rat (gray) and Mouse (black) in proximal dendritic fields. In the Y-axes, the percentages of colocalized PSD95 with respect to the total PSD95 signals were plotted, while the X-axes reports the different time points considered as DIV. **(C)** Quantitative degree of co-localization between PSD95 and SYN1 in distal dendritic fields. **(D)** Representative image of SYN1 (red panel) and Gephyrin (green panel) signals, and co-localization (merge panel) at DIV12 in rat and mouse hippocampal neurons **(E)** Quantitative data representing the degree of co-localization between the two synaptic proteins Rat (gray) and Mouse (black) in proximal dendritic fields. In the Y-axes, the percentages of colocalized Gephyrin with respect to the total Gephyrin signals were plotted, while the X-axes reports the different time points considered as DIV. **(F)** Quantitative degree of co-localization between Gephyrin and SYN1 in distal dendritic fields. **(G)** Rat and **(H)** mouse spontaneous electrical activity at different DIV. ^**^p ≤ 0.01 and ^*^p ≤ 0.05 (One-Way ANOVA with Holm-Sidak's pairwise multiple comparison).

To determine the time course of synaptogenesis, we immunostained neurons at DIV6, 9, 12 and 15 with antibodies against the presynaptic protein Synapsin-1 (SYN1) and against the postsynaptic protein PSD95 or Gephyrin. Co-localization of these two markers was used as an indicator of a mature excitatory synapse (Figure 4A). The co-localization was expressed as the percentage of PSD95 that was co-localized with SYN1, for primary dendrites it was 77% for rat and 89% for mouse cultures. Already at DIV6 for secondary dendrites it was 59% for rat and 94% for mouse, without significant changes at later stages. Primary apical dendrites of rat neurons showed significantly lower PSD95/SYN1 co-localization at DIV12 compared to mouse neurons (Figure 4B; ANOVA, *P* = 0.003). On the other hand, there was a significantly higher co-localization in secondary dendrites of rat neurons at DIV6, 9, and 12 compared to mouse neurons (Figure 4C, ANOVA, *P* = 0.041, *P* = 0.006, *P* = 0.023). Co-localization of Gephyrin and SYN1 was used as an indicator of a mature inhibitory synapse (Figure 4D). The co-localization varied between 50–62% for primary dendrites and 40–54% for secondary dendrites already by DIV6 (Figure 4E). Mouse and rat primary neurons showed a constant maturation trend from DIV6 to DIV15. Secondary apical dendrites of rat neurons showed slightly higher Gephyrin/SYN1 co-localization at DIV9 (*P* = 0.035) and DIV12 (*P* = 0.015) compared to mouse neurons (Figure 4F, ANOVA). A characterization of passive membrane properties of cultured neurons at different days *in vitro* by whole-cell recordings revealed a stable resting membrane potential in rat and mouse neurons between DIV3 and 12, with a progressive increase in membrane capacitance consistent with the increase in surface area of neurons. Reflecting the formation of a functional network through synaptic contacts, mouse neurons showed spontaneous bursts of action potentials already at DIV6, adopting a clearly organized pattern by DIV12 (Figure 4H, Table 1). On the other hand, rat neurons showed an organized firing pattern of spontaneous action potentials only at DIV12 (Figure 4G, Table 1).

**Table 1:** **Electrophysiological neuronal maturation**.

**RAT**	**Resting (mV)**	**Capacitance (pF)**	**Mouse**	**Resting (mV)**	**Capacitance (pF)**
DIV3	57.8 ± 5.98 (n = 6)	15.7 ± 1.19 (n = 6)	DIV3	47.17 ± 7.30 (n = 6)	19.8 ± 3.03 (n = 5)
DIV6	50.75 ± 1.49 (n = 4)	33.8 ± 3.81 (n = 4)	DIV6	66 ± 1.38 (n = 5)	30.7 ± 2.17 (n = 5)
DIV9	53.75 ± 6.61 (n = 5)	49.8 ± 11.99 (n = 5)	DIV9	60.57 ± 2.97 (n = 7)	43.14 ± 4.82 (n = 7)
DIV12	63.25 ± 0.48 (n = 4)	60.02 ± 13.89 (n = 4)	DIV12	60.6 ± 4 (n = 5)	70.55 ± 16.83 (n = 5)

*Passive membrane properties recorded in developing hippocampal neurons from rat and mouse. Spontaneous activity and passive membrane properties recorded in developing hippocampal neurons from rat and mouse*.

### Morphological characterization of *Mecp*2^−/*y*^ hippocampal neurons

**Figure 5:**
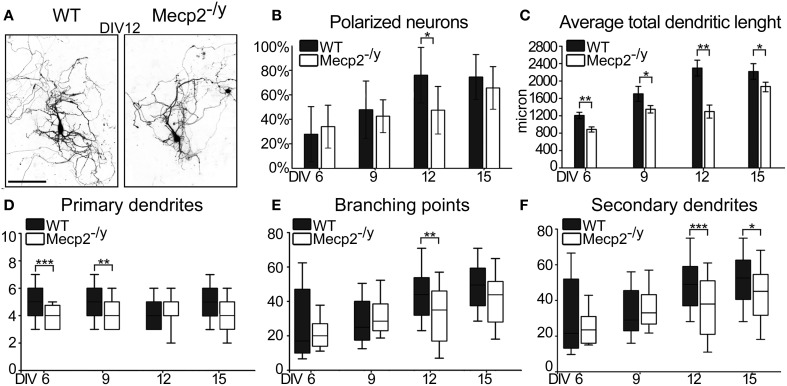
**Morphological characterization of WT and *Mecp2^−/*y*^* mouse developing hippocampal neurons**. **(A)** Representative images of both WT and *Mecp2*^−/*y*^ mouse hippocampal neurons at DIV12. (Scale Bar = 50 μm) **(B)** Quantification of relative amount of polarized neurons in culture Data are expressed as mean ± *SD*, *n* = 4 cultures **(C)** Quantitative data of the average total dendritic length. Data are expressed as mean ± s.e.m., *n* = 40. Quantification of primary dendrites **(D)**, branching points **(E)** and of the secondary dendrites (**F**) at DIV6, 9, 12, and 15. The median line of the boxes refers to the 50 percentile of the entire population. Mann-Whitney Rank Sum Test, ^***^P ≤ 0.001, ^**^P ≤ 0.01, ^*^P ≤ 0.05.

To determine how the lack of MeCP2 affects hippocampal neuronal development *in vitro*, we cultured neurons from male *Mecp2* knockout (*Mecp2*^−/*y*^**)** and wild type (WT) littermate mice. The hippocampi dissected out from each animal were plated separately, transfected with GFP and analyzed at DIV6, 9, 12, and 15. Compared to WT neurons, a lower proportion of *Mecp2*^−/*y*^ neurons specified an apical dendrite, and by DIV12 their developmental delay was significant (50% polarized neurons in *Mecp2*^−/*y*^ cultures vs. 77% in WT cultures; *P* = 0.020) (Figures 5A,B). During the following days (DIV12-15), dendritic polarization in *Mecp2*^−/*y*^ neurons reached levels comparable to that in WT neurons (69% polarized neurons in *Mecp2*^−/*y*^ cultures vs. 74% in WT cultures). The total dendritic length of *Mecp2*^−/*y*^ neurons was significantly smaller already at DIV6 compared to WT neurons (*Mecp2*^−/*y*^ 843 ± 87 μm vs. WT 1,206 ± 73 μm; *P* = 0.002). This difference remained over time *in vitro* and was much larger at DIV12 (*Mecp2*^−/*y*^ 1,426 ± 258 μm vs. WT 2,297 ± 181 μm; *P* = 0.007) (Figure 5C). To further understand the morphological features of *Mecp2*^−/*y*^ neurons, we analyzed primary and secondary dendrites, as well as branch points (Figures 5D,E,F). *Mecp2*^−/*y*^ neurons showed fewer primary dendrites at DIV6 and 9 compared to WT neurons (DIV6 *P* ≤ 0.001, DIV9 *P* = 0.002), but recovered at later stages (Figure 5D). The number of secondary dendrites and branch points were both comparable between WT and *Mecp2*^−/*y*^ neurons at the early time points considered (DIV6 and 9). However, at later stages of neuronal development (DIV12 and DIV15), the number of secondary dendrites and branch points were both significantly lower in *Mecp2*^−/*y*^ neurons than in WT neurons (DIV12 *P* ≤ 0.001) (Figures 5E,F).

### Spine density and morphology in *Mecp*2^−/*y*^ hippocampal neurons

**Figure 6:**
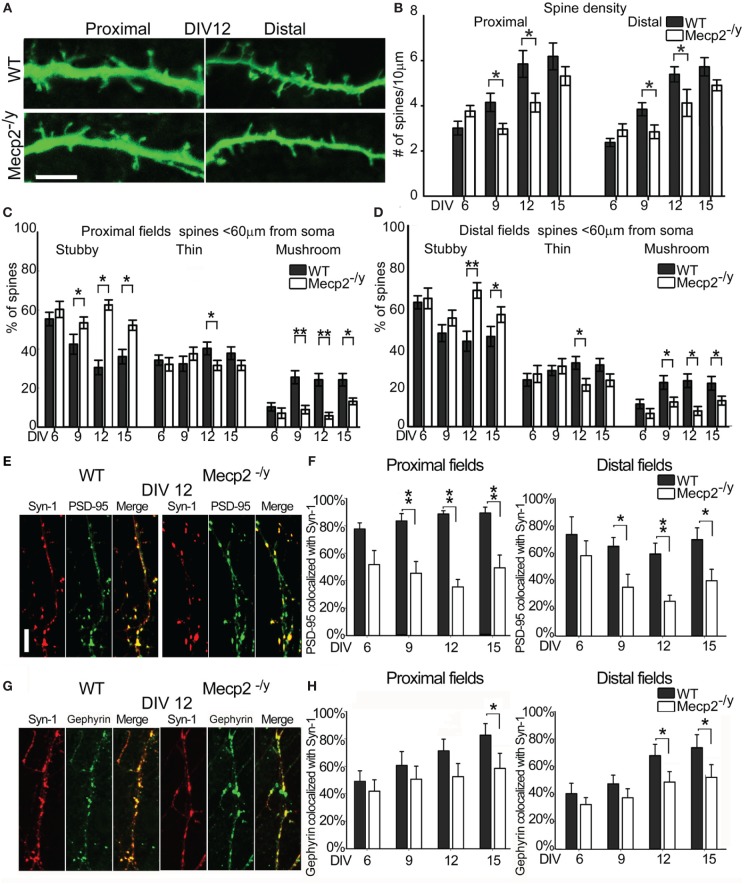
**Characterization of spines and synapses in WT and *Mecp2*^−/*y*^ mouse hippocampal neurons. (A)** Proximal and distal fields of WT and *Mecp2*^−/*y*^ mouse apical dendrites. (Scale Bar = 5 μm) **(B)** Spine density in proximal and distal region of WT (black) and *Mecp2*^−/*y*^ (white) apical dendrites at DIV6, 9,12, 15. **(C,D)** Spine type percentage with respect to total spine number at different time points (mean + standard errors). **(E)** Synapse identification by SYN1 (red) and PSD95 (green) signals co-localization (merge) at DIV12 in WT and MeCP2^−/*y*^ neurons (Scale bar = 5 μm). **(F)** Quantitative analysis of SYN/PSD95 co-localization in WT (black) and *Mecp2*^−/*y*^ (white) in proximal (left panel) and distal dendritic fields (right panel). The Y-axis reports the percentages of colocalized PSD95 with respect to total PSD95 signals, while the X-axis reports the different DIV considered. **(G)** Synapse identification by SYN1 (red) and Gephyrin (green) signals co-localization (merge) at DIV12 in WT and MeCP2^−/*y*^ neurons (Scale bar = 5 μm). **(H)** Quantitative analysis of SYN/ Gephyrin co-localization in WT (black) and *Mecp2*^−/*y*^ (white) in proximal (left panel) and distal dendritic fields (right panel). The Y-axis reports the percentages of colocalized Gephyrin with respect to total Gephyrin signals, while the X-axis reports the different DIV considered. ^**^p ≤ 0.01 and ^*^p ≤ 0.05 (One-Way ANOVA with Holm-Sidak's pairwise multiple comparison).

Dendritic spines on proximal primary apical dendrites and distal higher order dendrites of *Mecp2*^−/*y*^ and WT neurons expressing GFP were imaged and classified at different time points (Figure 6A). The spine density in proximal apical dendrites of WT neurons increased from 2.8 ± 0.3 spines/10 μm at DIV6 to 5.9 ± 0.6 spines/10 μm at DIV12, while in *Mecp2*^−/*y*^ neurons it went from 3.7 ± 0.3 spines/10 μm at DIV6 to 4.4 ± 0.6 spines/10 μm at DIV12; similar data were collected from distal dendrites (Figure 6B). *Mecp2*^−/*y*^ neurons had a lower spine density than WT neurons at DIV 9 and 12 (*P* = 0.014, *P* = 0.027), but spine density was comparable between the genotypes at DIV15. The percentage of stubby, thin and mushroom spine types in *Mecp2*^−/*y*^ neurons did not change over time, while WT neurons showed a progressive decrease in the percentage of stubby spines, and an increase in mushroom spines between DIV6 and DIV9 (Figure 6C). Consistently, the percentage of stubby spines in both proximal and distal fields at 12 and 15 DIV time points was significantly higher in *Mecp2*^−/*y*^ neurons (DIV12 proximal fields *Mecp2*^−/*y*^ 64 ± 2% vs. WT 36 ± 4%; *P* = 0.027; DIV12 distal fields *Mecp2*^−/*y*^ 76 ± 8% vs. WT 44 ± 5% *P* = 0.003), while the percentage of mushroom spines was significantly lower in *Mecp2*^−/*y*^ neurons (DIV12 proximal fields *Mecp2*^−/*y*^ 7 ± 2% vs. WT 24 ± 4%; *P* = 0.019; DIV12 distal fields *Mecp2*^−/*y*^ 9 ± 3% vs. WT 26 ± 5%; *P* = 0.043) (Figures 6C,D).

### Synaptogenesis in *Mecp*2^−/*y*^ hippocampal neurons

The percentage of co-localization between PSD95 and SYN1 was calculated in WT and *Mecp2*^−/*y*^ neurons from DIV6, 9, 12, and 15. While the percentage of co-localization of these markers of excitatory synapses was comparable in *Mecp2*^−/*y*^ and WT neurons at DIV6, all later stages were characterized by an overall significant difference. Specifically, the percentage of PSD95 co-localized with SYN1 was 50% lower in *Mecp2*^−/*y*^ neurons than in WT neurons in proximal and distal regions of the apical dendrite from DIV9 to DIV15 (DIV9 *P* = 0.005; DIV12 *P* = 0.004; *P* = 0.002; ANOVA) (Figures 6E,F). Figure 6E shows that in *Mecp2*^−/*y*^ neurons at DIV12, several PSD95 spots (green) were not co-localized with SYN1 (red) while all SYN1 spots are co-localized with PSD95. A further analysis of the density of SYN1 spots at DIV12 showed 4.3 ± 0.48 spots/10 μm in WT neurons and a decreased SYN1 density in *Mecp2*^−/*y*^ neurons with 3.2 ± 0.33 spots/10 μm (*P* = 0.025, ANOVA) while the density of PSD95 spots at DIV12 was not statistically different between WT and *Mecp2*^−/*y*^ neurons (WT 3,4 ± 0,28; *Mecp2*^−/*y*^ 3.1 ± 0.16). In the same way, the percentage of co-localization between Gephyrin and SYN1 was calculated in WT and *Mecp2*^−/*y*^ neurons from DIV6, 9, 12, and 15. The percentage of Gephyrin co-localization with SYN1 was 30% lower in *Mecp2*^−/*y*^ neurons than in WT neurons in proximal (at DIV 15, *P* = 0.047) and distal regions of the apical dendrite (at DIV12, *P* = 0.017 and DIV15, *P* = 0.023; ANOVA) (Figures 6G,H). Since the data of the co-localization analysis are expressed as the percentage of co-localized PSD95 or Gephyrin with respect to their total signals in dendrites, failure of co-localization between postsynaptic elements and SYN1 indicates the presence of a postsynaptic compartment that is not (or anymore) contacted by a presynaptic bouton.

## Discussion

In this study, we propose a revised staging system for the development of cultured hippocampal neurons from rat, and a new staging system for mouse neurons, both including 6 stages but with a slightly different time course between rat and mouse neurons. Our systematic description of each stage in hippocampal neuronal cultures using morphological and functional features allowed us to identify two breakpoints in the maturation of neurons from MeCP2 deficient mice, consisting in a delayed development of dendritic arborization from stage 4, and a reduction in established excitatory synapses at stages 5 and 6. These results indicate that both developmental and maintenance setbacks affect the final morphology and function of neurons in RTT individuals.

### Revision of the rat staging system

**Figure 7:**
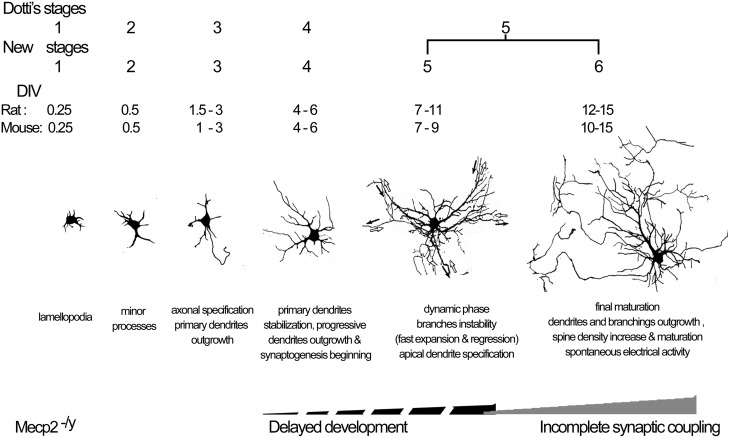
**Model of the *in vitro* neuron staging**. Model of the development of rat and mouse hippocampal neurons *in vitro*, and the timing of developmental and maintenance failure in *Mecp2*^−/*y*^ neurons.

The morphology of mature hippocampal pyramidal neurons is achieved over several days *in vitro*, but the current 5-stage developmental model for rat neuron cultures spans only from DIV1 to DIV7 (Dotti et al., 1988). Our systematic analysis of the events occurring from DIV1 to DIV15 indicated the necessity to expand the current model (reviewed by Caceres et al., 2012). We confirmed the early stages 1–2 at DIV < 1. In particular, we verified the recent observation by Dotti's group (Calderon de Anda et al., 2008) that shortly before axons become specified, neurons show a bipolar morphology (Figure 7). In our hands, Stage 3 started at DIV1.5 as in Dotti's model, but in addition, axonal specification was found to extend until DIV3, at which about 70% of neurons showed a Tau-1 positive/MAP-2 negative neurite (Figure 7). Stage 4, previously described as the “dendritic outgrowth” step that was limited to DIV4, can in fact be better defined as the time-period spanning DIV4-6, during which primary dendrites become stable, secondary dendrites show a progressive growth, and synapses start to appear (Figure 7). We propose to divide the old Stage 5, previously denominated “maturation,” in two different stages. Indeed, our data support the view that between DIV7-11 apical dendrites are established through a highly dynamic phase of dendritic expansion and regression, as suggested by Horton et al. (2006) (Figure 7). Thus, in our new model, Stage 5 corresponds to this dynamic phase while the actual maturation stage (now Stage 6) is characterized by a final step of dendritic growth and stabilization that spans between DIV12-15. Of note, these late time points have not been considered by any model in the literature. During Stages 5 and 6, spine density increased and, especially at the later DIVs of Stage 6, there was a shift in spine type composition with a reduction in the percentage of stubby and an increase in mushroom spine types. Recordings of membrane passive properties in rat neurons showed a progressive increase in membrane capacitance consistent with a progressive growth of dendrites, as described *in vivo* (Tyzio et al., 1999). The Vrest value recorded at DIV3 can be considered as quite low (−50 mV approximately), however these data are in accordance with previous studies focused on resting membrane potential at early developmental stages (Zhang, 2004). Moreover, the mean values cited in our paper are in line with those previously described for cultured pyramidal neurons (Coulter et al., 1992; Mangan and Kapur, 2004). Organized patterns of spontaneous action potential firing were observed only after DIV12, in line with data from previous studies using different culturing protocols (Bacci et al., 1999). Interestingly, excitatory synapses—estimated by the co-localization of pre- and postsynaptic markers—were already present at DIV6, much before spontaneous bursts of action potentials were detectable.

Our data are in agreement with a recent review article (Caceres et al., 2012), in which the 5 developmental stages were grouped into 3 polarization phases denominated “First phase of polarity: Generation of the first neurite” (Stage 1); “Second phase of polarity: Generation of the axon and dendrites from minor neurites” (Stage 2, 3); “Third phase: Axon–dendrite commitment” (Stages 4, 5). It remains to be determined whether our new Stage 6 should be included in Dotti's Phase III because at this time point dendrites and axons are already specified and therefore, strictly speaking, it should be considered as a maturation phase rather than a polarization phase.

### Comparison of rat and mouse neuronal development

The new staging system in 6 Stages proposed for rat cultures also applies for mouse neurons. Comparison between rat and mouse cultures however, highlighted some differences in their developmental time courses. In general, mouse neurons matured faster than rat neurons during the initial axon outgrowth and the final phase of dendrite maturation, and were more unstable during the central dynamic phase. In particular, mouse neurons started the final wave of dendritic growth already at DIV10 (Stage 6), while it occurred from DIV12 onward in rat neurons. In addition, mouse neurons showed more precocious spine maturation. Indeed, the increase in mushroom spines was already evident by Stage 5 in mouse neurons, while it occurred at stage 6 in rat neurons. In contrast, the dynamic phase leading to the establishment of an apical dendrite initially appeared to progress slowly in mouse neurons to reach similar values of rat neurons, from DIV9 onward. Furthermore, bursting patterns of spontaneous action potentials appeared at DIV6 in mouse cultures, while they were detected in rat cultures only at DIV12. We are not aware of such comparisons in dissociated neuronal cultures; however, a few studies are available *in vivo*. Anatomical and electrophysiological comparison of rat and mouse hippocampal newborn neurons showed that CA1 neurons had similar structure, with a few differences in dendritic morphology and membrane physiology, while their number is strictly related to the life span of each species (Routh et al., 2009; Amrein et al., 2011) and to different mouse strains (C57BL/6 vs. 129/SvEv) (Routh et al., 2009).

### Synapse maturation *in vitro*

Previous studies reported that GABAergic currents mediate the first synaptic activity appearing during development in various brain regions. Hippocampal sharp waves (SPW) bursts, represent the main hippocampal field pattern during the first postnatal days (Leinekugel et al., 2002), suggested to be the *in vivo* equivalents of Giant Depolarizing Potentials, GDPs, immature network patterns described *in vitro* in neonatal hippocampal slices, generated by depolarizing and excitatory GABA (Ben-Ari et al., 1989). A shift of GABA from the depolarizing to the hyperpolarizing direction occurs after the second postnatal week. Interictal discharges are generated by GABA receptor antagonists whose frequency increases with age (Le Magueresse et al., 2006). A developmental shift of the GABA signaling has been also observed in culture, shown to be promoted by the developmental increase in GABAergic activity (Ganguly et al., 2001). Within a week of the establishment of dissociated hippocampal cultures, spontaneous electrical activity is organized in bursting patterns (Kamioka et al., 1996; Cohen et al., 2008) promoted by a GABA-mediated excitatory drive (Ganguly et al., 2001; Waddell et al., 2011), as observed also in acute slices (Ben-Ari, 2002). After one week, synaptically- and intrinsically-mediated bursts characterize the spontaneous activity of cultured hippocampal neurons (Bacci et al., 1999). A blockade of GABAA receptors caused enhancement of the burst discharges (Cohen et al., 2008) without loss of synchronization (Bacci et al., 1999; Cohen et al., 2008); see also our Figure S1). We noted that TTX blocked the bursting activity, never observed in low density cultures (data not shown) revealing that synchronized bursting requires connectivity among a critical number of cells (Ivenshitz and Segal, 2010). It is not excluded that intrinsic conductances participate to characterize neuronal discharges observed in cultured cells.

### Developmental *vs*. maintenance failure in Rett syndrome

In order to clarify the pathogenetic mechanism underlying Rett syndrome, *Mecp2* null, mutant *Mecp2* knock-in, and conditional knockout mouse mutants have been generated (Calfa et al., 2011; Samaco and Neul, 2011; Li and Pozzo-Miller, 2012). All these models develop RTT-like clinical features, such as normal early postnatal development followed by a reduction in brain weight and neuronal cell size, as well as reduced volume of specific brain areas (Belichenko et al., 2008, 2009). In addition, abnormalities in the dendritic structure were detected in the dentate gyrus, CA1 and motor cortex (Belichenko et al., 2009), including lower spine density and smaller area of spine heads and spine neck length (Belichenko et al., 2009) although another study did not confirmed these findings (Chapleau et al., 2012). Landi et al., have recently described dendritic spine dynamics in the somatosensory cortex by 2-photon imaging in *Mecp2* mutant mice and time-lapse imaging revealed a dramatic effect of *Mecp2* deletion in short-term structural plasticity of cortical spines at ages (P25–26) slightly preceding the onset of symptoms (Landi et al., 2011). Indeed, the authors reported that “the density of dendritic spines and filopodia was reduced in mutant mice. Also the length of spines was slightly but significantly shorter when compared with controls” and suggested it could be largely due to a decreased fraction of filopodia and to their higher stability compared to WT controls (Landi et al., 2011). However, at later stages (P40), “spine motility was comparable in WT and MeCP2 null mice whereas spine density remains significantly impaired in MeCP2 mutants. Thus, the deletion of MeCP2 impairs spine motility during the critical period for cortical plasticity and synaptogenesis.” Another study investigated the role of MeCP2 in dendritic development in newborn granule neurons in the dentate gyrus showing that hippocampal neurogenesis was not affected by the absence of MeCP2, while immature dentate gyrus neurons in *Mecp2* knockout mice exhibited deficits in their ability to transition to later stages of development (Smrt et al., 2007). From the findings described above and those emerging from more recent investigations on induced pluripotent stem cells derived from RTT patients, the general consensus is that the lack of MeCP2 does not affect neurogenesis nor the early stages of neuronal development, but results in a slower maturation phase. However, the recapitulation of the main RTT-like features in adulthood using an inducible conditional knockout of *Mecp2* strongly suggested that proper MeCP2 function is required also for the maintenance of the neuronal phenotype (McGraw et al., 2011). Experiments to compare the electrophysiological properties of WT and KO Rett mouse were not performed. Further studies aimed to understand the origin of burst patterns will be executed in brain slices.

We found that *Mecp2* knockout neurons grow dendrites slower than wild type neurons, rather than suffering a retraction of mature dendritic arbors. We also observed in *Mecp2* knockout neurons a delayed developmental maturation of spine number, and morphology, which normally includes fewer stubby spines and more mushroom dendritic spines. Moreover, after a normal initial development, we found fewer excitatory and inhibitory synapses at later stages, suggesting increased synapse selection/elimination/pruning. In a recent study *in vitro* (Larimore et al., 2009), silencing the endogenous *Mecp2* gene did not affect axonal morphology but reduced total dendritic length, while overexpression of wild type human *MECP2* gene induced an increase in axonal and dendritic length and branching. Intriguingly, the overexpression of human *MECP2* carrying typical Rett syndrome missense mutations negatively affected dendritic branching and outgrowth as well as axonal length (Larimore et al., 2009). These studies suggest the possibility that different *MECP2* mutations may lead to either loss or gain of MeCP2 protein function.

Previous morphological studies in postmortem brain samples from RTT individuals described a decreased dendritic growth in pyramidal neurons (Armstrong et al., 1995) More recently Chapleau et al. (2009) confirmed in rodents brain slices that a transfection of mutated MeCP2 or silencing of endogenous MeCP2 were able to reduce dendritic spine density in neurons. The data collected in the present manuscript allowed us to confirm these results from human brains and *ex vivo* model. The presence of different “phenotypic checkpoints” (Ben-Ari and Spitzer, 2010) and the fact that different species present a differential timing (Khazipov and Luhmann, 2006) of neurodevelopmental features (i.e., apical dendrite specification and axonal outgrowth) reiterate the importance to have a specie-specific morphological signature of neurological disorder (Ben-Ari and Spitzer, 2010). In conclusion, the possibility to characterize the neuronal development *in vitro*, in a step by step mode is a peculiar characteristic of primary cell cultures but while they are considered a valuable research tool is important to remind that they represent a reductionistic model of the *in vivo* system suitable for high-throughput screening of new drugs.

In conclusion, the new staging system proposed here expands our current knowledge of the development of hippocampal neurons *in vitro* and provides novel insights in the pathogenesis of Rett syndrome. The staging system described here can be used to study the development of neurons from mouse models of other neurodevelopmental disorders and may pave the way to obtain more standardized cultures, which are highly needed for neurodevelopmental toxicological studies in order to obtain valuable substitutes to animal testing.

### Conflict of interest statement

The authors declare that the research was conducted in the absence of any commercial or financial relationships that could be construed as a potential conflict of interest.

## Supplementary material

Figure S1 **Representative traces of spontaneous electrical activity in rat neurons at DIV12**. Before (upper trace) and after (bottom trace) 10 min superfusion with Gabazine.

## References

[B1] Aibel L. Martin-Zanca D. Perez P. Chao M. V. (1998). Functional expression of TrkA receptors in hippocampal neurons. J. Neurosci. Res. 54, 424–431 10.1002/(SICI)1097-4547(19981101)54:3%3C424::AIDJNR13%3E3.0.CO;2-6 9819147

[B2] Akbarian S. (2002). Diseases of the mind and brain: Rett's syndrome. Am. J. Psychiatry 159, 1103 10.1176/appi.ajp.159.7.1103 12091185

[B3] Amir R. E. Van den Veyver I. B. Wan M. Tran C. Q. Francke U. Zoghbi H. Y. (1999). Rett syndrome is caused by mutations in X-linked MECP2, encoding methyl-CpG-binding protein 2. Nat. Genet. 23, 185–188 10.1038/13810 10508514

[B4] Amrein I. Isler K. Lipp H. P. (2011). Comparing adult hippocampal neurogenesis in mammalian species and orders: influence of chronological age and life history stage. Eur. J. Neurosci. 34, 978–987 10.1111/j.1460-9568.2011.07804.x 21929629

[B5] Armstrong D. Dunn J. K. Antalffy B. Trivedi R. (1995). Selective dendritic alterations in the cortex of Rett syndrome. J. Neuropathol. Exp. Neurol. 54, 195–201 10.1097/00005072-199503000-00006 7876888

[B6] Armstrong D. D. (2001). Rett syndrome neuropathology review 2000. Brain Dev. 23(Suppl. 1), S72–S76 10.1016/S0387-7604(01)00332-1 11738845

[B7] Bacci A. Verderio C. Pravettoni E. Matteoli M. (1999). Synaptic and intrinsic mechanisms shape synchronous oscillations in hippocampal neurons in culture. Eur. J. Neurosci. 11, 389–397 10.1046/j.1460-9568.1999.00440.x 10051739

[B8] Barnes A. P. Polleux F. (2009). Establishment of axon-dendrite polarity in developing neurons. Annu. Rev. Neurosci. 32, 347–381 10.1146/annurev.neuro.31.060407.125536 19400726 PMC3170863

[B9] Belichenko N. P. Belichenko P. V. Li H. H. Mobley W. C. Francke U. (2008). Comparative study of brain morphology in Mecp2 mutant mouse models of Rett syndrome. J. Comp. Neurol. 508, 184–195 10.1002/cne.21673 18306326

[B10] Belichenko P. V. Oldfors A. Hagberg B. Dahlstrom A. (1994). Rett syndrome: 3-D confocal microscopy of cortical pyramidal dendrites and afferents. Neuroreport 5, 1509–1513 10.1097/00001756-199407000-00025 7948850

[B11] Belichenko P. V. Wright E. E. Belichenko N. P. Masliah E. Li H. H. Mobley W. C. (2009). Widespread changes in dendritic and axonal morphology in Mecp2-mutant mouse models of Rett syndrome: evidence for disruption of neuronal networks. J. Comp. Neurol. 514, 240–258 10.1002/cne.22009 19296534

[B12] Ben-Ari Y. (2002). Excitatory actions of gaba during development: the nature of the nurture. Nat. Rev. Neurosci. 3, 728–739 10.1038/nrn920 12209121

[B13] Ben-Ari Y. Cherubini E. Corradetti R. Gaiarsa J. L. (1989). Giant synaptic potentials in immature rat CA3 hippocampal neurones. J. Physiol. 416, 303–325 2575165 10.1113/jphysiol.1989.sp017762 PMC1189216

[B14] Ben-Ari Y. Spitzer N. C. (2010). Phenotypic checkpoints regulate neuronal development. Trends Neurosci. 33, 485–492 10.1016/j.tins.2010.08.005 20864191 PMC2963711

[B15] Caceres A. Ye B. Dotti C. G. (2012). Neuronal polarity: demarcation, growth and commitment. Curr. Opin. Cell Biol. 24, 547–553 10.1016/j.ceb.2012.05.011 22726583 PMC3425660

[B16] Calderon de Anda F. Gartner A. Tsai L. H. Dotti C. G. (2008). Pyramidal neuron polarity axis is defined at the bipolar stage. J. Cell Sci. 121, 178–185 10.1242/jcs.023143 18187450

[B17] Calfa G. Percy A. K. Pozzo-Miller L. (2011). Experimental models of Rett syndrome based on Mecp2 dysfunction. Exp. Biol. Med. (Maywood) 236, 3–19 10.1258/ebm.2010.010261 21239731 PMC3059199

[B18] Chapleau C. A. Boggio E. M. Calfa G. Percy A. K. Giustetto M. Pozzo-Miller L. (2012). Hippocampal CA1 pyramidal neurons of Mecp2 mutant mice show a dendritic spine phenotype only in the presymptomatic stage. Neural Plast. 2012, 976164 10.1155/2012/976164 22919518 PMC3418521

[B19] Chapleau C. A. Calfa G. D. Lane M. C. Albertson A. J. Larimore J. L. Kudo S. (2009). Dendritic spine pathologies in hippocampal pyramidal neurons from Rett syndrome brain and after expression of Rett-associated MECP2 mutations. Neurobiol. Dis. 35, 219–233 10.1016/j.nbd.2009.05.001 19442733 PMC2722110

[B20] Cohen E. Ivenshitz M. Amor-Baroukh V. Greenberger V. Segal M. (2008). Determinants of spontaneous activity in networks of cultured hippocampus. Brain Res. 1235, 21–30 10.1016/j.brainres.2008.06.022 18602907

[B21] Coulter D. A. Sombati S. Delorenzo R. J. (1992). Electrophysiology of glutamate neurotoxicity *in vitro*: induction of a calcium-dependent extended neuronal depolarization. J. Neurophysiol. 68, 362–373 1388200 10.1152/jn.1992.68.2.362

[B22] Dotti C. G. Sullivan C. A. Banker G. A. (1988). The establishment of polarity by hippocampal neurons in culture. J. Neurosci. 8, 1454–1468 3282038 10.1523/JNEUROSCI.08-04-01454.1988 PMC6569279

[B23] Ehlers M. D. Polleux F. (2010). Neuronal and glial cell biology. Curr. Opin. Neurobiol. 20, 529–530 10.1016/j.conb.2010.06.004 20678922

[B24] Ganguly K. Schinder A. F. Wong S. T. Poo M. (2001). GABA itself promotes the developmental switch of neuronal GABAergic responses from excitation to inhibition. Cell 105, 521–532 10.1016/S0092-8674(01)00341-5 11371348

[B25] Gleeson J. G. Polleux F. (2012). Neurodevelopment and disease. Curr. Opin. Neurobiol. 22, 735–736 10.1016/j.conb.2012.07.010 22889697

[B26] Gordon-Weeks P. R. (1991). Evidence for microtubule capture by filopodial actin filaments in growth cones. Neuroreport 2, 573–576 10.1097/00001756-199110000-00005 1756237

[B26a] Guy J. Hendrich B. Holmes M. Martin J. E. Bird A. (2001). A mouse Mecp2-null mutation causes neurological symptoms that mimic Rett syndrome. Nat Genet 27, 322–326 11242117 10.1038/85899

[B27] Harris K. M. Jensen F. E. Tsao B. (1992). Three-dimensional structure of dendritic spines and synapses in rat hippocampus (CA1) at postnatal day 15 and adult ages: implications for the maturation of synaptic physiology and long-term potentiation. J. Neurosci. 12, 2685–2705 1613552 10.1523/JNEUROSCI.12-07-02685.1992 PMC6575840

[B28] Hirokawa N. (1994). Microtubule organization and dynamics dependent on microtubule-associated proteins. Curr. Opin. Cell Biol. 6, 74–81 10.1016/0955-0674(94)90119-8 8167029

[B29] Hirokawa N. Sato-Yoshitake R. Kobayashi N. Pfister K. K. Bloom G. S. Brady S. T. (1991). Kinesin associates with anterogradely transported membranous organelles *in vivo*. J. Cell Biol. 114, 295–302 10.1083/jcb.114.2.295 1712789 PMC2289077

[B30] Horton A. C. Yi J. J. Ehlers M. D. (2006). Cell type-specific dendritic polarity in the absence of spatially organized external cues. Brain Cell Biol. 35, 29–38 10.1007/s11068-006-9003-y 17940911

[B31] Ivenshitz M. Segal M. (2010). Neuronal density determines network connectivity and spontaneous activity in cultured hippocampus. J. Neurophysiol. 104, 1052–1060 10.1152/jn.00914.2009 20554850

[B32] Jacobs S. Nathwani M. Doering L. C. (2010). Fragile X astrocytes induce developmental delays in dendrite maturation and synaptic protein expression. BMC Neurosci. 11:132 10.1186/1471-2202-11-132 20955577 PMC2970604

[B33] Kaech S. Banker G. (2006). Culturing hippocampal neurons. Nat. Protoc. 1, 2406–2415 10.1038/nprot.2006.356 17406484

[B34] Kamioka H. Maeda E. Jimbo Y. Robinson H. P. Kawana A. (1996). Spontaneous periodic synchronized bursting during formation of mature patterns of connections in cortical cultures. Neurosci. Lett. 206, 109–112 10.1016/S0304-3940(96)12448-4 8710163

[B35] Kaufmann W. E. Moser H. W. (2000). Dendritic anomalies in disorders associated with mental retardation. Cereb. Cortex 10, 981–991 10.1093/cercor/10.10.981 11007549

[B36] Khazipov R. Luhmann H. J. (2006). Early patterns of electrical activity in the developing cerebral cortex of humans and rodents. Trends Neurosci. 29, 414–418 10.1016/j.tins.2006.05.007 16713634

[B37] Kim K. Y. Hysolli E. Park I. H. (2011). Neuronal maturation defect in induced pluripotent stem cells from patients with Rett syndrome. Proc. Natl. Acad. Sci. U.S.A. 108, 14169–14174 10.1073/pnas.1018979108 21807996 PMC3161557

[B38] Kossel A. H. Williams C. V. Schweizer M. Kater S. B. (1997). Afferent innervation influences the development of dendritic branches and spines via both activity-dependent and non-activity-dependent mechanisms. J. Neurosci. 17, 6314–6324 9236241 10.1523/JNEUROSCI.17-16-06314.1997 PMC6568345

[B39] Landi S. Putignano E. Boggio E. M. Giustetto M. Pizzorusso T. Ratto G. M. (2011). The short-time structural plasticity of dendritic spines is altered in a model of Rett syndrome. Sci. Rep. 1, 45 10.1038/srep00045 22355564 PMC3216532

[B40] Larimore J. L. Chapleau C. A. Kudo S. Theibert A. Percy A. K. Pozzo-Miller L. (2009). Bdnf overexpression in hippocampal neurons prevents dendritic atrophy caused by Rett-associated MECP2 mutations. Neurobiol. Dis. 34, 199–211 10.1016/j.nbd.2008.12.011 19217433 PMC2726722

[B41] Leinekugel X. Khazipov R. Cannon R. Hirase H. Ben-Ari Y. Buzsaki G. (2002). Correlated bursts of activity in the neonatal hippocampus *in vivo*. Science 296, 2049–2052 10.1126/science.1071111 12065842

[B42] Le Magueresse C. Safiulina V. Changeux J. P. Cherubini E. (2006). Nicotinic modulation of network and synaptic transmission in the immature hippocampus investigated with genetically modified mice. J. Physiol. 576, 533–546 10.1113/jphysiol.2006.117572 16901939 PMC1890366

[B43] Li W. Pozzo-Miller L. (2012). Beyond widespread Mecp2 deletions to model Rett syndrome: conditional spatio-temporal knockout, single-point mutations and transgenic rescue mice. Autism. (Suppl.1), 5, 01–05 10.4172/2165-7890.S1-005 23946910 PMC3740402

[B44] Mandell J. W. Banker G. A. (1996). A spatial gradient of tau protein phosphorylation in nascent axons. J. Neurosci. 16, 5727–5740 8795628 10.1523/JNEUROSCI.16-18-05727.1996 PMC6578967

[B45] Mangan P. S. Kapur J. (2004). Factors underlying bursting behavior in a network of cultured hippocampal neurons exposed to zero magnesium. J. Neurophysiol. 91, 946–957 10.1152/jn.00547.2003 14534286 PMC2892720

[B46] Matijevic T. Knezevic J. Slavica M. Pavelic J. (2009). Rett syndrome: from the gene to the disease. Eur. Neurol. 61, 3–10 10.1159/000165342 18948693

[B47] Matus A. (1991). Microtubule-associated proteins and neuronal morphogenesis. J. Cell Sci. Suppl. 15, 61–67 10.1242/jcs.1991.Supplement_15.9 1824108

[B48] McGraw C. M. Samaco R. C. Zoghbi H. Y. (2011). Adult neural function requires MeCP2. Science 333, 186 10.1126/science.1206593 21636743 PMC3150190

[B49] Mewes A. Franke H. Singer D. (2012). Organotypic brain slice cultures of adult transgenic P301S mice–a model for tauopathy studies. PLoS ONE 7:e45017 10.1371/journal.pone.0045017 22984603 PMC3439393

[B50] Routh B. N. Johnston D. Harris K. Chitwood R. A. (2009). Anatomical and electrophysiological comparison of CA1 pyramidal neurons of the rat and mouse. J. Neurophysiol. 102, 2288–2302 10.1152/jn.00082.2009 19675296 PMC2775381

[B51] Samaco R. C. Neul J. L. (2011). Complexities of Rett syndrome and MeCP2. J. Neurosci. 31, 7951–7959 10.1523/JNEUROSCI.0169-11.2011 21632916 PMC3127460

[B52] Saud K. Arriagada C. Cardenas A. M. Shimahara T. Allen D. D. Caviedes R. (2006). Neuronal dysfunction in Down syndrome: contribution of neuronal models in cell culture. J. Physiol. Paris 99, 201–210 10.1016/j.jphysparis.2005.12.013 16646156

[B53] Saywell V. Viola A. Confort-Gouny S. Le Fur Y. Villard L. Cozzone P. J. (2006). Brain magnetic resonance study of Mecp2 deletion effects on anatomy and metabolism. Biochem. Biophys. Res. Commun. 340, 776–783 10.1016/j.bbrc.2005.12.080 16380085

[B54] Schule B. Armstrong D. D. Vogel H. Oviedo A. Francke U. (2008). Severe congenital encephalopathy caused by MECP2 null mutations in males: central hypoxia and reduced neuronal dendritic structure. Clin. Genet. 74, 116–126 10.1111/j.1399-0004.2008.01005.x 18477000

[B55] Scott E. K. Luo L. (2001). How do dendrites take their shape? Nat. Neurosci. 4, 359–365 10.1038/86006 11276225

[B56] Sharma P. Ando D. M. Daub A. Kaye J. A. Finkbeiner S. (2012). High-throughput screening in primary neurons. Methods Enzymol. 506, 331–360 10.1016/B978-0-12-391856-7.00041-X 22341232 PMC3564665

[B57] Smith C. C. Vedder L. C. Mcmahon L. L. (2009). Estradiol and the relationship between dendritic spines, NR2B containing NMDA receptors, and the magnitude of long-term potentiation at hippocampal CA3-CA1 synapses. Psychoneuroendocrinology 34(Suppl. 1), S130–S142 10.1016/j.psyneuen.2009.06.003 19596521 PMC2796081

[B58] Smrt R. D. Eaves-Egenes J. Barkho B. Z. Santistevan N. J. Zhao C. Aimone J. B. (2007). Mecp2 deficiency leads to delayed maturation and altered gene expression in hippocampal neurons. Neurobiol. Dis. 27, 77–89 10.1016/j.nbd.2007.04.005 17532643 PMC2789309

[B59] Subramaniam B. Naidu S. Reiss A. L. (1997). Neuroanatomy in Rett syndrome: cerebral cortex and posterior fossa. Neurology 48, 399–407 10.1212/WNL.48.2.399 9040729

[B60] Tyzio R. Represa A. Jorquera I. Ben-Ari Y. Gozlan H. Aniksztejn L. (1999). The establishment of GABAergic and glutamatergic synapses on CA1 pyramidal neurons is sequential and correlates with the development of the apical dendrite. J. Neurosci. 19, 10372–10382 10575034 10.1523/JNEUROSCI.19-23-10372.1999 PMC6782402

[B61] Urbanska M. Blazejczyk M. Jaworski J. (2008). Molecular basis of dendritic arborization. Acta Neurobiol. Exp. (Wars) 68, 264–288 18511961 10.55782/ane-2008-1695

[B62] Waddell J. Kim J. Alger B. E. McCarthy M. M. (2011). The depolarizing action of GABA in cultured hippocampal neurons is not due to the absence of ketone bodies. PLoS ONE 6:e23020 10.1371/journal.pone.0023020 21886776 PMC3158756

[B63] Wu G. Y. Zou D. J. Rajan I. Cline H. (1999). Dendritic dynamics *in vivo* change during neuronal maturation. J. Neurosci. 19, 4472–4483 10341248 10.1523/JNEUROSCI.19-11-04472.1999 PMC6782592

[B64] Zhang Z. W. (2004). Maturation of layer V pyramidal neurons in the rat prefrontal cortex: intrinsic properties and synaptic function. J. Neurophysiol. 91, 1171–1182 10.1152/jn.00855.2003 14602839

