# Supplementary figures and images for: Developmental and maintenance defects in Rett syndrome neurons identified by a new mouse staging system in vitro
Source: Front Cell Neurosci. 2014 Feb 5;8:18. doi: 10.3389/fncel.2014.00018 (PMC3914021; doi:10.3389/fncel.2014.00018)

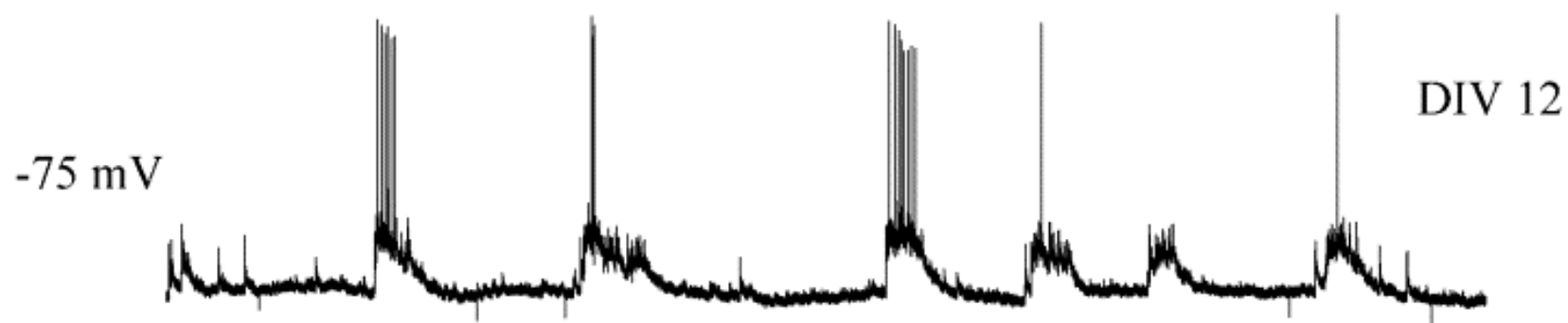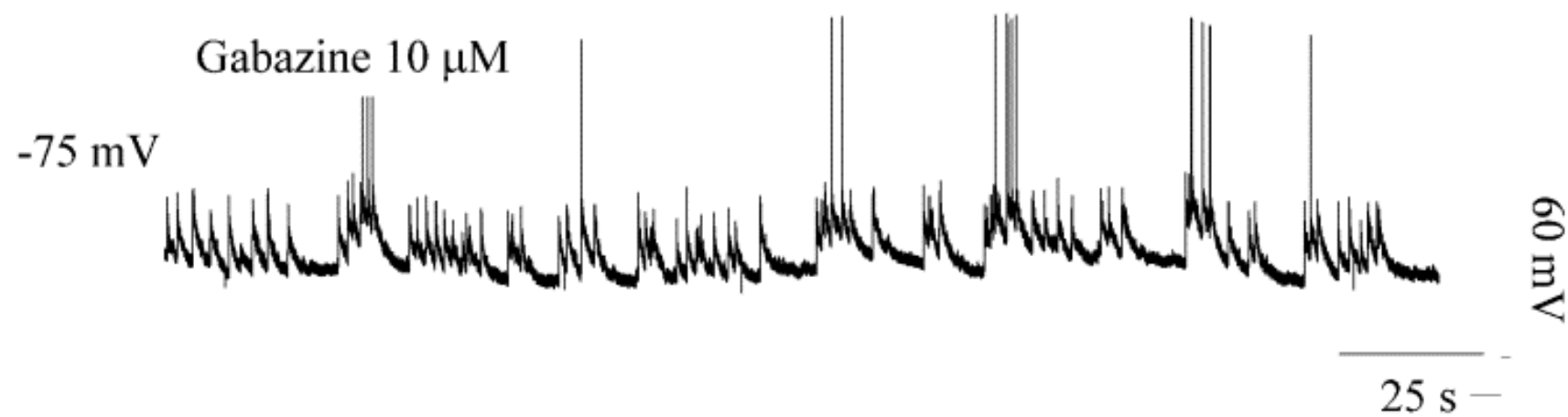

Supplement: Figure S1 — Representative traces of spontaneous electrical activity in rat neurons at DIV12. Before (upper trace) and after (bottom trace) 10 min superfusion with Gabazine. [file DataSheet1.PDF]
